# Chalcones Isolated from *Arrabidaea brachypoda* Flowers as Inhibitors of NorA and MepA Multidrug Efflux Pumps of *Staphylococcus aureus*

**DOI:** 10.3390/antibiotics9060351

**Published:** 2020-06-20

**Authors:** Luís Mário Rezende-Júnior, Leila Maria de Sousa Andrade, Antonio Linkoln Alves Borges Leal, Avilnete Belem de Souza Mesquita, Ana Lurdes Portela de Araújo dos Santos, José de Sousa Lima Neto, José Pinto Siqueira-Júnior, Carlos Emídio Sampaio Nogueira, Glenn William Kaatz, Henrique Douglas Melo Coutinho, Natália Martins, Cláudia Quintino da Rocha, Humberto Medeiros Barreto

**Affiliations:** 1 Laboratory of Research in Microbiology, Federal University of Piaui, Teresina, Piaui 64049-550, Brazil; mariorezende@ufpi.edu.br (L.M.R.-J.); leila.andrade@ifpi.edu.br (L.M.d.S.A.); antoniolinkoln@hotmail.com (A.L.A.B.L.); avilnete@yahoo.com.br (A.B.d.S.M.); 2 Laboratory of Advanced Studies in Phytomedicines, Federal University of Maranhão, São Luís, Maranhão 65080-805, Brazil; analurdes-portela@hotmail.com (A.L.P.d.A.d.S.); claudiarocha3@yahoo.com.br (C.Q.d.R.); 3 Laboratory of Organic Geochemistry, Federal University of Piaui, Teresina, Piauí 64049-550, Brazil; limaneto5@gmail.com; 4 Laboratory of Genetics of Microorganisms, Federal University of Paraiba, Joao Pessoa, Paraiba 58051-900, Brazil; jpsiq@uol.com.br; 5 Department of Biological Chemistry, Regional University of Cariri, Crato, Ceará 63105-010, Brazil; carlos.emidio@globo.com; 6 Department of Medicine, Division of Infectious Diseases, Wayne State University School of Medicine, Detroit, MI 48201, USA; gkaatz@juno.com; 7 Laboratory of Microbiology and Molecular Biology, Regional University of Cariri, Crato, Ceará 64049-550, Brazil; hdmcoutinho@gmail.com; 8 Faculty of Medicine, University of Porto, Alameda Prof. Hernâni Monteiro, 4200-319 Porto, Portugal; 9 Institute for Research and Innovation in Health (i3S), University of Porto, 4200-135 Porto, Portugal

**Keywords:** *Arrabidaea brachypoda*, Chalcone, antimicrobial activity, efflux pumps, efflux pump inhibitors, resistance to antibiotics

## Abstract

Bacterial resistance to antibiotics has become a public health issue around the world. The present study aimed to evaluate the antibacterial activity of chalcones isolated from flowers of *Arrabidaea brachypoda*, and their potential as efflux pump inhibitors of *Staphylococcus aureus* efflux pumps. Microdilution assays were performed with natural products from *A. brachypoda.* Chalcones **1**, **3**, **4,** and **5** did not show intrinsic antimicrobial activity against all *S. aureus* strains tested, but they were able to potentiate the Norfloxacin action against the SA1199-B (*norA*) strain, with a better modulating action for the **4** trimethoxylated chalcone. All chalcones were also able to potentiate the action of EtBr against SA1199-B strain, suggesting a potential NorA inhibition. Moreover, chalcone **4** was able to interfere in the activity of MepA, and interfered weakly in the *QacA/B* activity. Molecular docking analyzes showed that tested chalcones are capable of binding in the hydrophobic cavity of NorA and MepA, in the same Norfloxacin binding site, indicating that chalcone **4** compete with the antibiotic for the same NorA and MepA binding sites. Association of chalcone **4** with Norfloxacin could be an alternative against multidrug resistant *S. aureus* over-productive of NorA or MepA.

## 1. Introduction

The indiscriminate use of antibiotics has created conditions for the accelerated development of multidrug resistant bacteria [1]. Infections caused by coagulase positive *Staphylococcus* spp., which were previously controlled by β-lactam antibiotics, have been reported in a raising frequency [2,3]. Methicillin-resistant *S. aureus* (MRSA) strains have progressively acquired different resistance mechanisms, reducing the chances of achieving therapeutic success [4]. The high prevalence of MRSA infections comes from the spread of some clones, with only five clones described worldwide, descendants of a single *S. aureus* ancestral strain that acquired the chromosomal gene *mecA* encoding the transpeptidase PBP2’ which has a low affinity for Methicillin [5]. On the other side, the widespread use of fluoroquinolones as an alternative therapy against MRSA infections has led to the development of bacterial resistance to these drugs [6]. In some cases, the glycopeptide Vancomycin is the only therapeutic option for the treatment of *MRSA* infections, despite its nephrotoxic and ototoxic effects [7]. MRSA strains remains one of the main causes of hospital-acquired and community-acquired infections, being classified as a high priority resistant pathogen that need of new therapeutic alternatives [8].

Resistance to antibiotics can be acquired by different mechanisms, among these, it is worth to highlight the antibiotic extrusion by transmembrane proteins, known as efflux pumps [9,10]. These proteins consist of extrusion systems that give bacteria resistance to multiple drugs and can be present in both Gram-positive [11] and Gram-negative bacteria [12]. Several efflux pumps present in *S. aureus* strains have been identified, such as NorA, NorB, NorC, MdeA, SepA, MepA, SdrM, and LmrS efflux pumps, which are encoded by the bacterial chromosome, while the QacA/B, QacG, QacH, QacJ and Smr are encoded by plasmid [13]. In view of this scenario, several researches have been looking for new technological strategies, such as the discovery of new antimicrobial agents [14], or new compounds capable of inhibiting resistance mechanisms [15]. In this context, efflux pump inhibitors (EPIs) have been proposed as a technological strategy against multidrug resistant bacteria overexpressing efflux pump genes [16,17].

*Arrabidaea brachypoda* (Bignoniaceae), popularly known as “cipó-uma”, “tintureiro” or “Cervejinha do campo”, is a native bush of the Brazilian cerrado, abundantly branched, presenting an average height between 1.0 and 2.0 m with leaves and flowers in terminal influences in pinkish-purple color [18]. *A. brachypoda* roots and leaves are widely used in traditional medicine in southeastern and northeastern Brazil for kidney disease and joint pain [19,20].

Chalcones consist of a class of open-chain flavonoids with the basic structure of 1,3-diphenyl-2-propen-1-one [21]. This class has attracted a great scientific interest throughout history due to its wide range of bioactivities, including antioxidant, anticancer, antidiabetic, anti-inflammatory, antimicrobial, anti-tuberculosis effects, among others [20,21,22,23,24]. It is also known that chalcones can trigger programmed cell death in eukaryotes, as demonstrated in the protozoan *Plasmodium falciparum* [24].

In fact, it has already been demonstrated that the ethanolic extract obtained from the flowers of *A. brachypoda*, as well as its dichloromethane fraction and the isolated compound Brachydin B were able to increase the antibiotic Norfloxacin activity against a *S. aureus* strain over-productive of NorA efflux pump [25]. Based on the popular use of *A. brachypoda* and growing concern with the advancement of bacterial resistance to currently available antimicrobials, it is relevant to continue studies on the antimicrobial properties of this plant, especially its flowers, considering their possible activities of resistance modulation to antibiotics of its isolated compounds. Thus, the present study aimed to assess the antimicrobial activity of chalcones isolated from *A. brachypoda* flowers, as well as their ability to enhance the antibiotics activity against different *S. aureus* strains over-productive of efflux pumps.

## 2. Results and Discussion

### 2.1. Compound Identification and Evaluation of the Antimicrobial Activity

**Figure 1:**
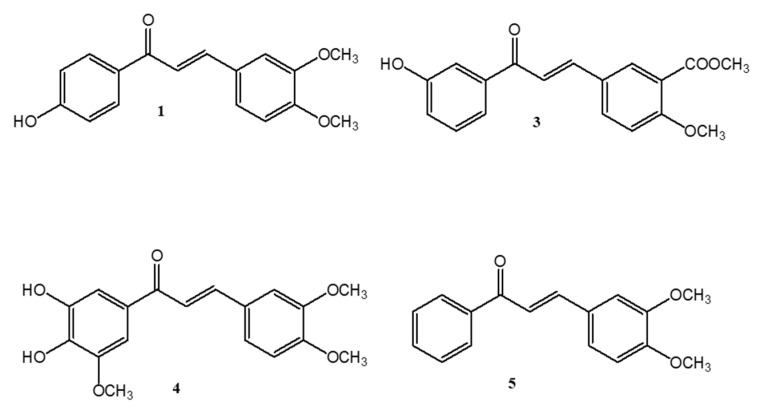
Chemical structure of 4’-Hydroxy-3-4-dimethoxy-chalcone (**1**), 3’-Hydroxy-3-acetate, 4-methoxy-chalcone (**3**), 3’,4’-dihydroxy, 3,4,4’-trimethoxy-chalcone (**4**) and 3,4-dimethoxy-chalcone (**5**) isolated from *A. brachypoda* flowers.

In the dichloromethane fraction from *A. brachypoda* flowers, 4 chalcones were identified (Figure 1) and their chemical features are presented in Table 1 and Table 2. Chalcones class has attracted great scientific interest throughout history due to its wide range of biological activities, such as antitumor and antiparasitic activities [26]. Considering the flexibility of its chemical production, synthetic chalcones have been extensively investigated [27].

Mass spectrometry (MS) and absorption spectroscopy in the UV-Vis region have been widely employed for the structural characterization of chalcones. Based on experiments using the electron spray ionization (ESI) method, through using mass spectrometry equipment of low resolution and high-resolution mass spectrometry (The spectrum in S1), it was possible to propose the structure of the compounds. For all isolated chalcones, very similar spectra were observed in the ultraviolet region at around 360 nm had strong UV absorption at the bond. The UV spectra and mass fragmentation data were compared with spectral data from commercial standard from our research group.

As shown in Table 1, MS/MS experiments proved some of the suspected fragmentation pathways, including support for sequential losses. In negative ion ESI-MS/MS, all target compounds yielded prominent [M − H]^−^ ions. Some common features, such as loss of CH_3_, H_2_0 and CO_2_, were observed in the MS/MS spectra, and were consistent with the literature. The [M − H− CH_3_]^−^ ion was a characteristic fragment of chalcones. In addition, [M – H − 2CH_3_]^−^ fragments were observed for dimethoxylated chalcones. Therefore, the loss of one or two CH_3_ could be adopted to identify single- or multi-methoxylated chalcones in negative ion ESI-MS/MS. Loss of CO and CO_2_ from [M − H]^−^ ions was attributed to the structure of the C-ring.

Several studies have demonstrated the importance of chalcones in plants’ defense against phytopathogenic fungal species [28,29,30]. Chalcones have been reported as antifungal agents and as inhibitors of fungal biofilms formation [28,31]. Natural products from plants have been considered as clinically irrelevant when they show MIC values above 1000 µg/mL [32]. MIC values found for chalcones tested are presented in Table 3. These results indicate that chalcones isolated from *A. brachypoda* were inactive against all *S. aureus* strains.

### 2.2. Evaluation of the Modulating Effect of Isolated Chalcones on the Resistance to Norfloxacin

**Figure 2:**
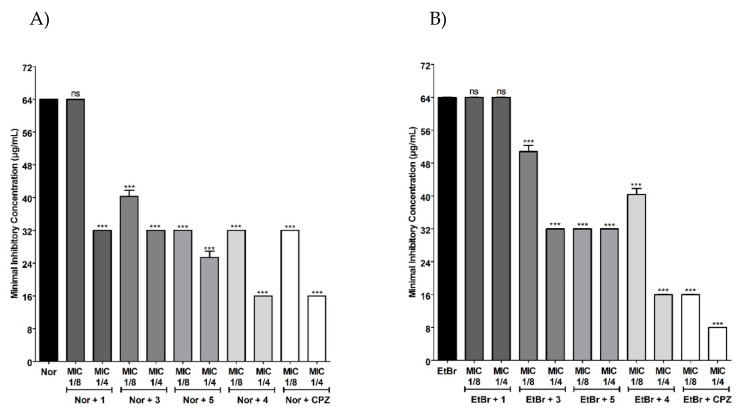
MIC values of (**A**) Norfloxacin (*Nor*) and (**B**) Ethidium Bromide (EtBr) in the absence or presence of chalcones **1**, **3**, **5**, **4** or Chlorpromazine (CPZ) against *S. aureus* SA1199-B (overexpressing *norA* gene). Each result represents the geometric mean of three simultaneous experiments. Differences found between treatment with Norfloxacin or EtBr alone or associated with chalcones or CPZ were analyzed through one-way analysis of variance (ANOVA) followed by Bonferroni post-test and were considered statistically significant when *p* < 0.05. *** *p* < 0.0001.

The Norfloxacin-resistance modulating activity was initially evaluated using the SA1199-B strain, resistant to Norfloxacin by overexpression of the *norA* gene [33]. Results showed that all chalcones increased the Norfloxacin activity against SA1199-B strain (Figure 2A). Among these, the chalcone **4** demonstrated the best modulating effect, decreasing the MIC values from 64 to 16 μg/mL (4-fold reduction), followed by chalcones **5**, **3** and **1**. Modulating effect showed by chalcone **4** was similar to that verified for the known NorA inhibitor, Chlorpromazine (CPZ), suggesting that chalcone **4** could be a promisor efflux pump inhibitor (EPI).

To test this hypothesis, modulation assays were conducted using Ethidium Bromide (EtBr) instead Norfloxacin, once the unique mechanism of resistance known to EtBr is by efflux pumps [34]. Chalcones **3**, **4** and **5** were also able to modulate the EtBr resistance in SA1199-B strain (Figure 2B). These results indicate that the modulating effect presented by these compounds, mainly by chalcone **4**, could be related with its action as NorA inhibitors, leading to EtBr accumulation, as well as Norfloxacin, into the bacterial cell. NorA is a member of the Major Facilitator Superfamily (MFS) belonging to the proton-driven antiport group [11]. NorA inhibitors have been considered as enhancers of the antimicrobials’ effectiveness, such as fluoroquinolones when tested in combination [35,36,37].

**Figure 3:**
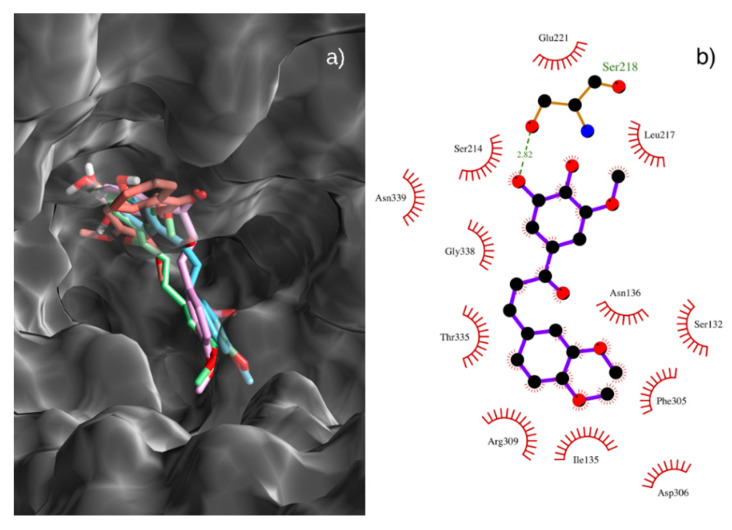
(**a**) Best poses of chalcones **1** (tinted blue), **3** (pink), **4** (green) and **5** (orange) on the binding site of the NorA model. (**b**) 2D ligand-protein interaction diagram of chalcone **4** and the NorA protein model. Contacts depicted in green.

Molecular docking between isolated chalcones and NorA were performed for analysis and confirmation of this interaction. Figure 3A shows the best poses of the 4 tested substances docked on the binding site of the NorA model. Out of the 4, chalcone **4** displayed the best binding score of −7.8 kcal/mol, followed by chalcones **5**, **3,** and **1**, with affinities of −7.5, −7.5 and −7.1 kcal/mol, respectively. This is in tune with the EPI assays previously discussed. All tested molecules roughly interacted with the same residues. Figure 3B displays the 2D ligand-protein interaction map of chalcone **4**. A hydrogen bond with residue Ser218 is depicted in green and this is the reason for chalcone **4** obtaining the best docking score. Chalcone **4** also interacts with other residues on the binding site, such as Ile135, Thr335 and Asn339 between other amino acid residues.

Based on these results, one could argue that these molecules hinder the binding of Norfloxacin to the binding site of the NorA protein, and thus act as NorA competitive inhibitors. To further test this hypothesis, we docked the Norfloxacin antibiotic to the NorA model. Through analysis of the interaction map it was stated that Norfloxacin binds roughly to the same region of the model binding site, interacting with residues Thr335, Asn339, Ser336, Ile135, between other amino acid residues.

NorA inhibition by chalcones was strongly influenced by the methoxylation degree, since chalcone **4** is trimethoxylated and showed the better modulating activity. The presence of methoxy groups has been reported as a factor that favors the interaction of a compound with NorA [38]. Another previous study found that the addition of methoxyl groups increased the lipophilicity of the studied flavanones, as well as that the tetramethoxylated compound had a greater modulatory effect on Norfloxacin resistance in SA1199-B than other di- or trimethoxylated compounds [39].

Modulation tests with chalcone **4** against different *S. aureus* strains overproducing TetK, MsrA, MepA, QacA/B or QacC efflux pumps were carried out to analyze the activity of chalcone **4** on these *S. aureus* efflux pumps, thus expanding their possibilities of further study and therapeutic use. Results showed that chalcone **4** weakly interfered in the QacA/B activity and did not inhibit the activity of the TetK, MsrA and QacC efflux pumps (Table 4). On the other side, chalcone **4** was able to interfere in the activity of MepA reducing the MIC values of both Norfloxacin and EtBr against the K2068 strain. MepA is a member of the multidrug and toxin extrusion (MATE) superfamily, which is dependent of H^+^ or Na^+^ gradient to extrude Norfloxacin or EtBr [10].

**Figure 4:**
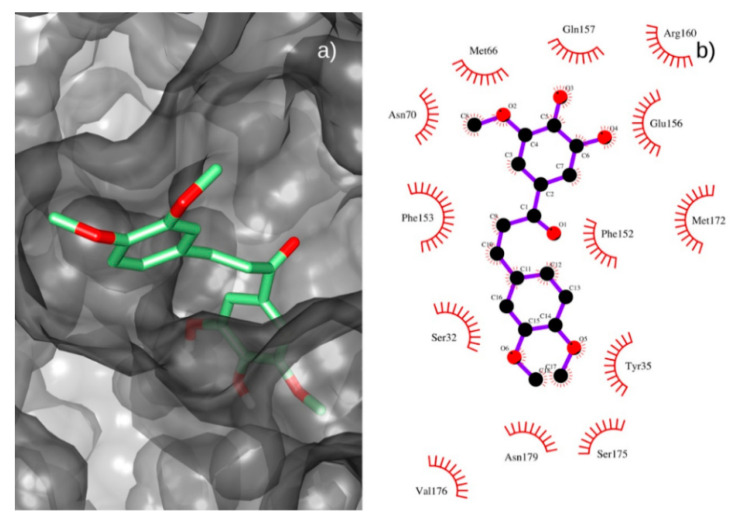
(**a**) Best pose of chalcone **4** (green) on the binding site of the MepA model. (**b**) 2D ligand-protein interaction diagram of chalcone **4** and the MepA protein model.

Docking of chalcone **4** against the MepA model is shown in Figure 4A. Additionally, a 2D ligand-protein interaction diagram of chalcone **4** and the MepA protein model is provided in Figure 4B.

Chalcone **4** interacted with several residues on MepA model’ binding site. Close contacts were stablished with Ser32, Met66, Val176, Asn179, Ser175, Tyr35, Ser32, Phe153, Phe152, Met172, Glu156, Asn70 and Gln157 residues. Thus, chalcone **4** showed a good positioning to hinder the antibiotics binding to the MepA protein, thus corroborating the results of modulating assays found for *S. aureus* K2068 overexpressing *mepA.*

These results indicate that chalcone **4** is a modulating agent for Norfloxacin resistance in *S. aureus* strains overexpressing *norA* and *mepA* genes, acting as an EPI. Therefore, this compound could be used in combination with Norfloxacin to enhance the activity of this antibiotic against resistant *S. aureus* strains by overproduction of efflux pumps, such as NorA and MepA.

## 3. Materials and Methods

### 3.1. Plant Material

Plant material was collected in the cerrado regions of the Brazilian state of Minas Gerais, in the municipality of João Pinheiro (latitude 17°44’33’’ South and longitude 46°10’21” Western) in 2016. The flowers were dried in a circulate air oven at 40 °C and crushed in a knife mill. The plant was identified in the Herbarium José Badine of the Federal University of Ouro Preto, Minas Gerais, Brazil, by the botanist Dr. Maria Cristina Teixeira Braga Messias (Voucher 17935). Permission to collect the botanical material used in the present study was granted by the National System of Management of Genetic Heritage and Associated Traditional Knowledge (SISGEN), Genetic Heritage Management Council, Ministry of Environment of Brazil under registration number: A3D296E.

### 3.2. Preparation of the Ethanolic Extract and Obtaining the Diclorometanic Fraction

Dried flowers (500 g) were macerated with ethanol at room temperature. Crude ethanolic extract was obtained after filtration and evaporated to dryness under vacuum at approximately 40 °C, lyophilized for 48 h yielding 60 g of ethanolic extract. A portion of the obtained extract (6.2 g) was weighed and dissolved in 100 mL of water: methanol solution (7:3). Subsequently, 25 mL of dichloromethane was used to incorporate into the previously prepared solution, obtaining two solution phases. This procedure was performed 3 times, using a 24-h interval between each collection. The dichloromethane fraction was dried with the aid of a drier.

### 3.3. Isolation and Identification of Chalcones

Dichloromethane fraction (1.5 g) was purified on a glass column, filled with an adsorbent, silica gel 60 (0.063–0.200 mm). The column was packed with hexane and then the sample was injected on top of the column. A gradient of increasing polarity of solvents was used as the mobile phase hexane/ethyl acetate and ethyl acetate/methanol. In total, 300 fractions were obtained, which were then grouped by thin layer chromatography according to the similarity of the profile. They were weighed and analyzed by HPLC-UV/vis, HRMS and data compared with literature. This procedure allowed the isolation of 4 compounds from the dichloromethane fraction.

### 3.4. Compound Identification

Mass spectrometry experiments were performed on LCQ Fleet equipment (Thermo Scientific) equipped with a direct sample insertion device via flow injection analysis (FIA). The studied matrix was analyzed by electrospray ionization (ESI), fragmentation in multiple stages (MS^2^, MS^3^ and MS^n^) was carried out in an ion prison interface (IT). The positive mode was selected for the generation and analysis of mass spectra for first order (MS) and for the other multistep experiments under the following conditions: capillary voltage, 25 V; Spray voltage, 5 kV; and capillary temperature, 275 °C. A carrier gas (N_2_) with a flow of 8 arbitrary units (A.U.), and the collision gas was helium (He). The acquisition of the range was *m/z* 100–2000. Xcalibur software version 1.3 (Thermo Finnigan, Waltham, MA, USA) was used to acquire and process the data.

High resolution electrospray ionisation mass spectrometry (HRESIMS) data were obtained with a Micromass LCT Premier time-of-flight mass spectrometer using an electrospray ionization (ESI) interface (Waters, Milford, MA, USA). The QTOF instrument was operated with an electrospray ion source (DualESI) in positive ion mode. Nitrogen at 350 °C, flow rate 10 L min^−1^ was used as drying gas. The nebulizer pressure was 40 psi, the capillary voltage 3500 V, the skimmer voltage 65 V, and the fragmentor voltage 100 V. In MS mode, the quadrupole was adjusted to total ion current whereas in MS/MS mode the quadrupole was adjusted to isolate only precursor ions with bandwidth Δ*m/z* = 1.3. MS/MS spectra were recorded after CID of precursor ions with a collision energy of 15 eV in a hexapole collision cell filled with nitrogen (99.999%). Ions were scanned in the mass ranges 100–1700 amu in MS mode and 50–500 amu in MS–MS mode. The TOF detector was operated in EDR (2 GHz) mode; the rate of acquisition was 1 spectrum s^−1^.

### 3.5. Strains and Drugs

Tests for checking intrinsic antimicrobial activity of compounds extracted from *A. brachypoda* flowers were performed against standard microbial strains: *S. aureus* ATCC 25923, *Escherichia coli* ATCC 25,922 and *Candida albicans* ATCC 10231. Moreover, chalcones were tested against the *S. aureus* strains overexpressing efflux pumps: SA1199-B (*norA* gene) [33], IS-58 (*tetK* gene) [40], RN-4220 (*msrA* gene) [41], K2068 (*mepA* gene) [42], K4114 (*qacA/B* gene) [43] and *S aureus* K4100 (*qacC* gene) [44]. Bacterial strains were maintained on Brain Infusion Heart agar (BHIA, Himedia, India) at 4 °C, and before the assay the cells were grown overnight at 37 °C in Brain Heart Infusion (BHI, Himedia, India). The antibiotics Norfloxacin (Nor), Tetracycline (Tet), Erythromycin (Ery), and the dye Ethidium Bromide (EtBr), as well as Chlorpromazine (CPZ) were obtained from Sigma Chemical Corp., St. Louis. With the exception of Nor that was dissolved in a mixture of 1 M NaOH and sterile distilled water (1:9 proportion), Tet, Eri, EtBr and CPZ were dissolved in sterile water.

### 3.6. Evaluation of the Antimicrobial Activity Assays

Stock solutions of chalcones (10,000 μg/mL) were prepared in DMSO followed by dilution in sterile water to a final concentration of 1024 μg/mL. Minimal inhibitory concentrations (MICs) were determined by micro-dilution assay [45] with modification in which the Muller-Hinton Broth was replaced by BHI broth. Bacterial suspensions of approximately 5 × 10^5^ CFU/mL were exposed to test-solutions 2-fold serial diluted (concentrations ranging from 8 to 512 μg/mL). Microtiter plates were incubated at 37 ºC for 24 h, then 20 μL of resazurin (0.01% *w/v* in sterile distilled water) was added to each well to detect bacterial growth by color change from blue to pink. MIC was defined as the lowest concentration at which no bacterial growth was observed. Antifungal assays were performed by micro-dilution method in SDB double-concentrated with yeast suspension of approximately 5 × 10^5^ CFU∙mL^−1^ and compound ranging from 8 to 512 μg/mL. Microtiter plates were incubated at 37 °C for 24 h.

### 3.7. Modulation of the Antibiotic Resistance Assays

To evaluate if chalcones were able to enhance the antibiotic activity against *S. aureus* overexpressing efflux pumps, the MICs of the antibiotics were determined in the presence or absence of sub-inhibitory concentrations of each natural product (1/8 or 1/4 MIC, respectively). Antibiotic concentrations ranged from 0.125 to 128 μg/mL. Microtiter plates were incubated at 37 °C for 24 h and readings were performed with resazurin as previously described. To verify if the drug-resistance modulation occurred due to EPI, modulation assays were performed by replacing antibiotics by EtBr, a known efflux pumps substrate [34], here used as an indicator of EPI. Control assays were also performed replacing chalcones by CPZ, a known EPI [46].

### 3.8. Docking Procedure

NorA model for the docking procedure was created as follows. Firstly, the NorA sequence of *S. aureus* SA1199B strain was retrieved from the Universal Protein Resource database (Uniprot, Entry Q03325). Then, the SWISS-MODEL service was used to build the homology model [47]. Out of the 50 templates generated, the one with the best global model quality estimation (GMQE) score was based on *E. coli* YajR transporter (PDB-ID: 3wdo) structure. The model was then uploaded to the MDWeb service, where it was solvated and submitted to a 0.5ns Molecular Dynamics run using the AMBER forcefield [48]. MepA model was generated by retrieving the protein sequence for the NCTC 8325 strain from the Uniprot database. The same procedure, as described above, was followed. The template of the multidrug and toxic compound extrusion (MATE) transporter of the *Bacillus halodurans* (PDB-ID: 5C6N) was chosen for the homology model.

For the docking procedure, which was carried out using the Autodock Vina [49] software (Scripps Research Institute, Jupiter—FL, USA), the grid box was defined as an 80Å × 80Å × 80Å box around the geometrical center of the model. Partial Gasteiger charges were added to ligand atoms, non-polar hydrogen atoms were mixed, while all other parameters were kept at their default values. The bst results were chosen based on the binding score.

### 3.9. Statistical Analysis

Each experiment was performed in triplicate and the results were normalized by calculating the geometric mean of the MICs. The standard error and standard deviation of the geometric mean were determined. Statistical analyzes were performed using the GraphPad Prism program, version 7.00. The occurrence of differences between treatment with antibiotics (or EtBr) alone or associated with chalcones tested (or CPZ) were examined through the one-way analysis of variance (ANOVA). Differences mentioned above were analyzed by the Bonferroni post-test and were considered statistically significant when *p* < 0.05.

## 4. Conclusions

Isolated chalcones **1**, **4** and **5** did not show intrinsic antimicrobial activity against *S. aureus*, *E. coli* and *C. albicans*; however, the chalcone **3** was active against *C. albicans.* In addition, all isolated compounds were able to potentiate the antibiotic activity of Nor against *S. aureus* SA1199-B and K2068 strains overproducers of NorA and MepA efflux pumps, respectively, being obtained a better modulating action for the chalcone **4** trimethoxylated. Modulating assays with EtBr and molecular docking of chalcone **4** with NorA or MepA indicated that this compound is a modulating agent for Nor resistance in *S. aureus* strains overexpressing *norA* and *mepA* genes, acting as an EPI. Therefore, this compound could be used in combination with Nor to treat infections caused by resistant *S. aureus* by overproduction of NorA or MepA.

## Figures and Tables

**Table 1:** Multistage MS^n^ data of chalcones by FIA-ESI/MS ion trap mass spectroscopy.

Chalcones (Exact Mass)	*m/z*; MS^n^ (% Base Peak)
283.0980	283[M − H]^−^; MS^2^[268]: 253(12), 239(100)
311.0919	311[M − H]^−^; MS^2^[253]: 28(100), 269(100)
329.1025	329[M − H]^−^; MS^2^[269]: 135(100); MS^3^[135]: 193(100)
267.1021	267[M − H]^−^; MS^2^[252]: 221(100)

**Table 2:** MS and HRESIMS data for compounds from *Arrabidaea brachypoda.*

Compound	Molecular Formula	Acurate Mass (amu)	Exact Mass	[M − H]^−^	Δm (ppm)
1	C_17_H_16_O_4_	283.0958	283.0980	283	2.2
3	C_18_H_16_O_5_	311.0918	311.0919	311	1.0
4	C_18_H_18_O_6_	329.1028	329.1025	329	−3.0
5	C_17_H_16_O_3_	267.1019	267.1021	267	2.0

**Table 3:** Geometric means of MIC values found for chalcones isolated from the flowers of *Arrabidaea brachypoda* against *S. aureus* strains overexpressing efflux pump genes, except ATCC 25,923 that is a standard strain (All values represent the geometric mean of three simultaneous experiments).

	MIC (μg/mL)			
Microbial Strains	1	3	4	5
*Staphylococcus aureus* ATCC 25923	1024	1024	1024	1024
*Staphylococcus aureus* IS-58	1024	1024	1024	1024
*Staphylococcus aureus* RN-4220	1024	1024	1024	1024
*Staphylococcus aureus* SA1199-B	1024	1024	1024	1024
*Staphylococcus aureus* K2068	1024	1024	1024	1024
*Staphylococcus aureus* K4414	1024	1024	1024	1024
*Staphylococcus aureus* K4100	≥1024	≥1024	≥1024	≥1024

**Table 4:** Minimum Inhibitory Concentrations (MICs) for antibiotics and Ethidium Bromide (EtBr) in the presence or absence of chalcones isolated from *Arrabidaea brachypoda* flowers against different *S. aureus* strains overproducing efflux pumps.

Efflux Pump Superfamily	Strain (Drug)	MIC (μg/mL)		
		Antibiotic/EtBr Alone	+ Chalcone 4 (MIC 1/8)	+ Chalcone 4 (MIC 1/4)
TetK (MFS)	IS-58 (Tet)	64	64 (0,0) *	64 (0,0)
	IS-58 (EtBr)	16	8 (2.0)	4 (4.0)
MsrA (ABC)	RN4220 (Ery)	32	32 (0,0)	32 (0,0)
	RN4220 (EtBr)	32	4 (8.0)	4 (8.0)
MepA (MATE)	K2068 (Nor)	4	2 (2.0)	1 (4.0)
	K2068 (EtBr)	8	4 (2.0)	4 (2.0)
QacA/B (MFS)	K4414 (BrEt)	32	32 (0,0)	16 (2.0)
QacC (SMR)	K4100 (BrEt)	128	128 (0,0)	128 (0,0)

ABC, ATP-Binding Cassette; Ery, Erythromycin; EtBr, Ethidium Bromide; MATE, Multi-Antimicrobial Extrusion Protein; MFS, Major Facilitator Superfamily; Nor, Norfloxacin; SMR, Small Multidrug Resistance; Tet, Tetracycline; * MIC reduction factor. Each result represents the geometric mean of three simultaneous experiments.
